# HIV-1 Nef Induces CCL5 production in astrocytes through p38-MAPK and PI3K/Akt pathway and utilizes NF-kB, CEBP and AP-1 transcription factors

**DOI:** 10.1038/srep04450

**Published:** 2014-03-24

**Authors:** Xun Liu, Ankit Shah, Mohitkumar R. Gangwani, Peter S. Silverstein, Mingui Fu, Anil Kumar

**Affiliations:** 1 Division of Pharmacology and Toxicology, School of Pharmacy; 2 Department of Basic Medical Science, School of Medicine, School of Medicine, University of Missouri-Kansas City, Kansas City, MO 64108

## Abstract

The prevalence of HIV-associated neurocognitive disorders (HAND) remains high in patients infected with HIV-1. The production of pro-inflammatory cytokines by astrocytes/microglia exposed to viral proteins is thought to be one of the mechanisms leading to HIV-1- mediated neurotoxicity. In the present study we examined the effects of Nef on CCL5 induction in astrocytes. The results demonstrate that CCL5 is significantly induced in Nef-transfected SVGA astrocytes. To determine the mechanisms responsible for the increased CCL5 caused by Nef, we employed siRNA and chemical antagonists. Antagonists of NF-κB, PI3K, and p38 significantly reduced the expression levels of CCL5 induced by Nef transfection. Furthermore, specific siRNAs demonstrated that the Akt, p38MAPK, NF-κB, CEBP, and AP-1 pathways play a role in Nef-mediated CCL5 expression. The results demonstrated that the PI3K/Akt and p38 MAPK pathways, along with the transcription factors NF-κB, CEBP, and AP-1, are involved in Nef-induced CCL5 production in astrocytes.

HIV-1 enters the CNS early in infection and has been demonstrated to cause a broad spectrum of neurological pathologies collectively known as HIV-associated neurocognitive disorders (HAND)^1^. The incidence of the most severe form of HAND, HIV-associated dementia (HAD), has declined because of the advent of effective antiretroviral therapy. However, the less severe form of HAND, minor cognitive motor disorder (MCMD) remains a significant problem in the era of highly active antiretroviral therapy (HAART)^2^.

Although HIV-1 does not infect neurons, the neurological effects of HIV-1 infection of the CNS are thought to be due to both the direct and indirect effects of viral infection. The direct effects of CNS infection with HIV-1 are due to the neurotoxicity of HIV-1 and HIV-1 proteins, including gp120, Tat and Nef, whereas indirect neurotoxicity is caused by the secretion of toxic mediators such as quinolinic acid and arachidonic acid metabolites, as well as pro-inflammatory cytokines which are released by microglia or astrocytes that are either infected with HIV-1 or exposed to HIV-1 proteins^3^,^4^,^5^. Approximately 70% of the brain is comprised of astrocytes, and these cells are subject to a low level of productive infection as well as non-productive infection with HIV-1^6^,^7^. As these cells are responsible for maintaining homeostasis in the brain, they play a major role in mediating the neurotoxic effects of HIV-1 infection of the CNS.

HIV-1 Nef is a multifunctional viral accessory protein of 27–35 kd that is abundantly expressed early in infection and has been shown to play an important role in numerous aspects of viral pathogenesis. The role of Nef in infected T-cells includes down-regulation of CD4, MHC-I and MHC-II, as well as enhancement of viral replication and virion infectivity (reviewed in^8^,^9^). The first studies that demonstrated the importance of Nef in HIV-1 pathogenesis focused on the Sydney blood bank cohort (SBBC). The SBBC was comprised of a group of patients who had been infected by blood transfusions from a single HIV-positive donor. The first report regarding the SBBC identified this group as long-term survivors of HIV-1 infection who were either long term nonprogressors or slow progressors^10^. Subsequent reports determined that the virus present in the donor as well as in the recipients had a common deletion in the nef/LTR region of the HIV-1 genome^11^. A subsequent study described progressive deletions in nef that suggested evolution towards the minimal nef/LTR sequence necessary for viral replication^12^. Results obtained with the SIV model of HIV/AIDS demonstrated that a functional nef gene plays a role in maintaining high viral loads and maximal pathogenic potential early in infection^13^. However, other studies have demonstrated that a nef-deleted virus may be pathogenic under certain circumstances. For example, a nef-deleted mutant of simian immunodeficiency virus (SIV) was fatal to 50% of macaque neonates^14^. Furthermore, a clone of SIVmac239 which was deleted in nef, vpr and a negative regulatory element, proved to be pathogenic in adult macaques when such determinations were made several years after inoculation^15^.

Although Nef is commonly found in the serum of AIDS patients^16^, fewer studies have investigated the presence of Nef in the cerebrospinal fluid of HIV-1 infected individuals or the functions of Nef in neuroinflammation caused by HIV-1. However, the presence of HIV-1 Nef mRNA and protein in astrocytes has been demonstrated in brain sections of individuals with AIDS-associated neuropathology^17^,^18^,^19^. Nef has been shown to increase leukocyte infiltration into the CNS along with the release of soluble factors such as CCL2, IL-6, TNF-α and IFN-γ^20^,^21^. Moreover, extracellular Nef is directly toxic to human neurons when added to culture media^22^.

CCL5, or RANTES (Regulated upon Activation, Normal T-cell Expressed, and Secreted), is a β chemokine and induces leukocyte migration by binding to either CCR1, CCR3 or CCR5. Elevated levels of CCL5 can mediate inflammatory responses and have been associated with a variety of inflammatory disorders^23^. CCL5 may also play a role in HAD because increased levels of the chemokine have been observed in the CSF of HIV-1 patients with HAD^24^. Furthermore, CCL5 has been shown to play a role in other neurodegenerative diseases including multiple sclerosis and Parkinson's Disease^25^,^26^.

In the present study we characterized the role of HIV-1 Nef in the induction of CCL5 in astrocytes. Furthermore, we determined the mechanisms responsible for the Nef-mediated increase of CCL5. Pharmacological inhibitors and siRNA were employed to determine the roles of the NF-κB, AP-1, C/EBP, PI3K-Akt and p38 MAPK pathways in the induction of CCL5 by Nef.

## Results

### HIV-1 Nef induces a time-dependent CCL5 up-regulation

**Figure 1:**
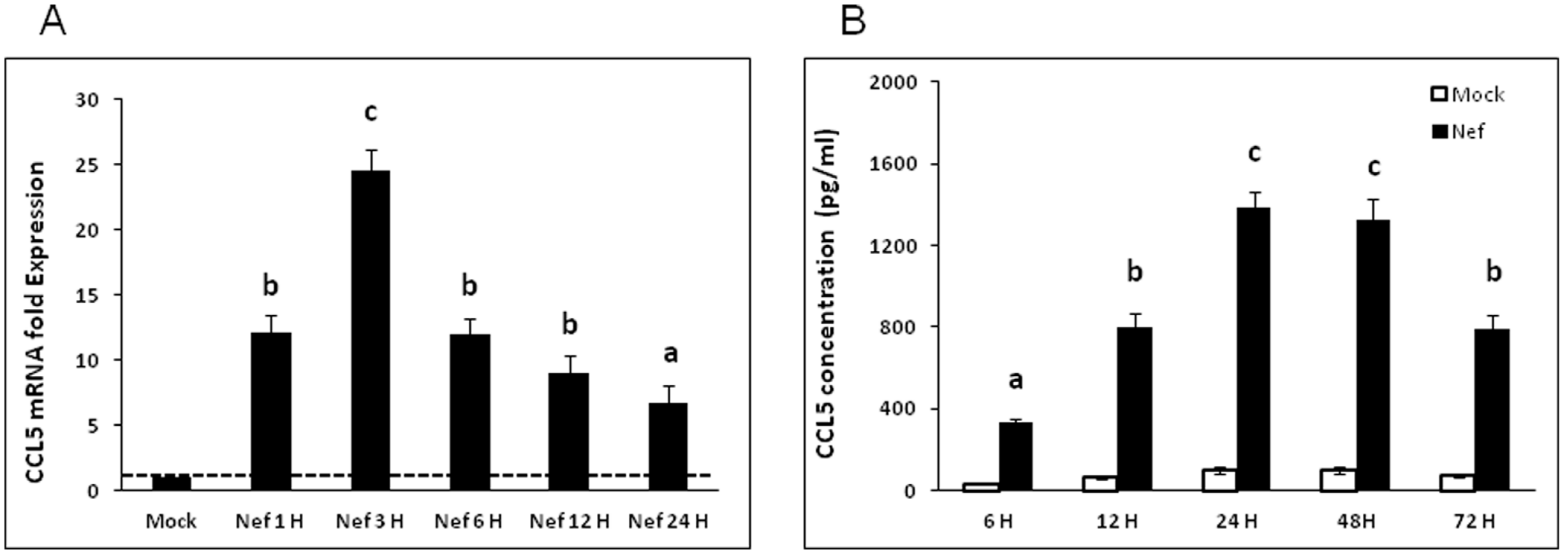
HIV-1 Nef induces CCL5 in SVGA astrocytes in a time-dependent manner. 7 × 10^5^ SVGA astrocytes were seeded in a 6-well plate and were transfected with a plasmid encoding HIV-1 Nef for 5 h using Lipofectamine 2000™. (A) The cells were harvested at 1 h, 3 h, 6 h, 12 h and 24 h for total RNA isolation and the expression of the CCL5 mRNA were determined by real time RT-PCR. Data in figures show fold-change relative to the mock-transfected wells at each time point. (B) CCL5 concentrations released in the supernatants were measured at 6 h, 12 h, 24 h, 48 h and 72 h by multiplex cytokine assay. Each of the bars represents mean ± SE of three independent experiments in triplicates Statistical analyses were performed using a one way ANOVA with an LSD post-hoc test. Treatments that are significantly different from each other (i.e. p < 0.05) are labeled with different letters.

Elevated levels of CCL5 have been detected in the CSF of patients suffering from various forms of HAND. Although it has been shown that Nef can induce CCL5 in microglia^31^, it is not known whether Nef has the same capabilities in astrocytes. SVGA, an immortalized astrocytic cell line, was transfected with a plasmid encoding nef and cells were harvested for mRNA and supernatants were collected to measure CCL5 protein. At all time points from 1 h to 24 h after transfection, CCL5 mRNA levels were significantly higher in nef-transfected cells than in the mock-transfected controls. At 1 h post-transfection the CCL5 mRNA level was ~12-fold higher than in mock-transfected cells. The mRNA level of CCL5 mRNA peaked at 3 h post-transfection, at which time the nef-transfected cells expressed CCL5 at levels that were 24.6 ± 1.6 fold higher than that in mock-transfected controls (Fig. 1A), but declined to ~12-fold at 6 h post-transfection. CCL5 protein levels were determined using Bio-Plex assays and while the level of CCL5 protein in nef-transfected cells was higher than that in mock-transfected cells starting at 6 h post-transfection, the peak level of CCL5 protein induced by Nef was at 24 hours post-transfection at which time the concentration of CCL5 in the supernatant of nef-transfected cells was 1381.8 ± 79.7 pg/ml (Fig. 1B). The level of CCL5 protein at 72 h post-transfection was still significantly higher than mock-transfected controls or the nef-transfected cells at 6 h post-transfection.

**Figure 2:**
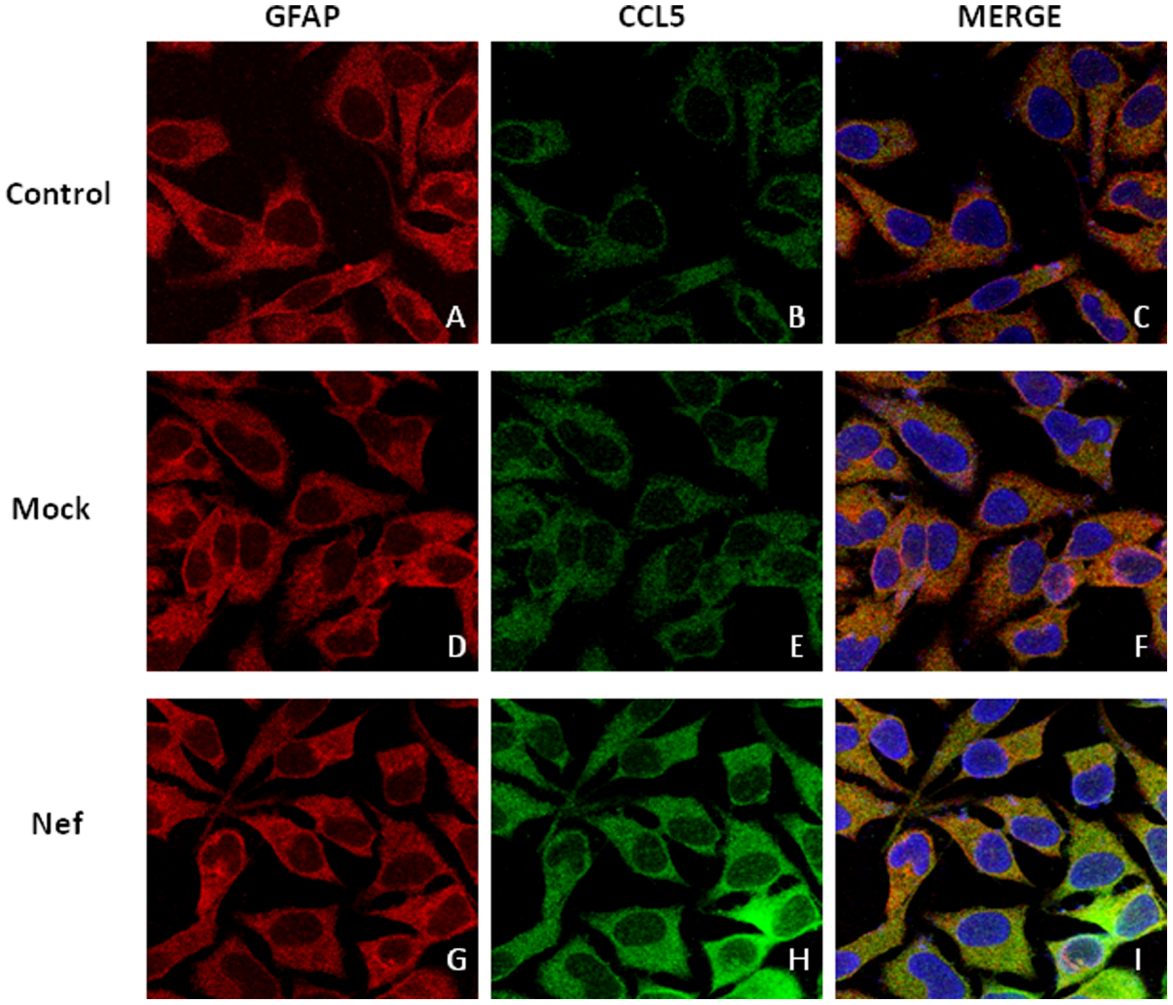
Confocal microscopy of CCL5 induced by HIV-1 Nef in astrocytes. 7 × 10^5^ SVGA astrocytes were seeded on a cover slip in a 6-well plate and were either mock-transfected (D–F) or transfected with a plasmid encoding HIV-1 Nef (G–I) for 5 h using Lipofectamine 2000™. Untreated controls (A–C) were used to visualize basal expression of CCL5 in astrocytes. 12 h later, cells were incubated with primary antibodies against CCL5 and GFAP and appropriate secondary antibodies labeled with Alexafluor 488 (CCL5) and Alexafluor 555 (GFAP). As illustrated in the figure, cells were stained for nucleus (blue); CCL5 (green) and GFAP (red) and the images were captured using Leica TCS SP5 II fluorescent microscope with 40× zoom oil emersion lens.

To confirm our findings regarding CCL5 induction by Nef, cells that were transfected with the plasmid encoding nef, as well as mock-transfected cells, were immunostained with fluorescent antibodies against CCL5 and appropriate secondary antibodies after which fluorescence was detected by confocal microscopy (Fig. 2).

### NF-κB pathway is involved in Nef-mediated increase of CCL5 expression

**Figure 3:**
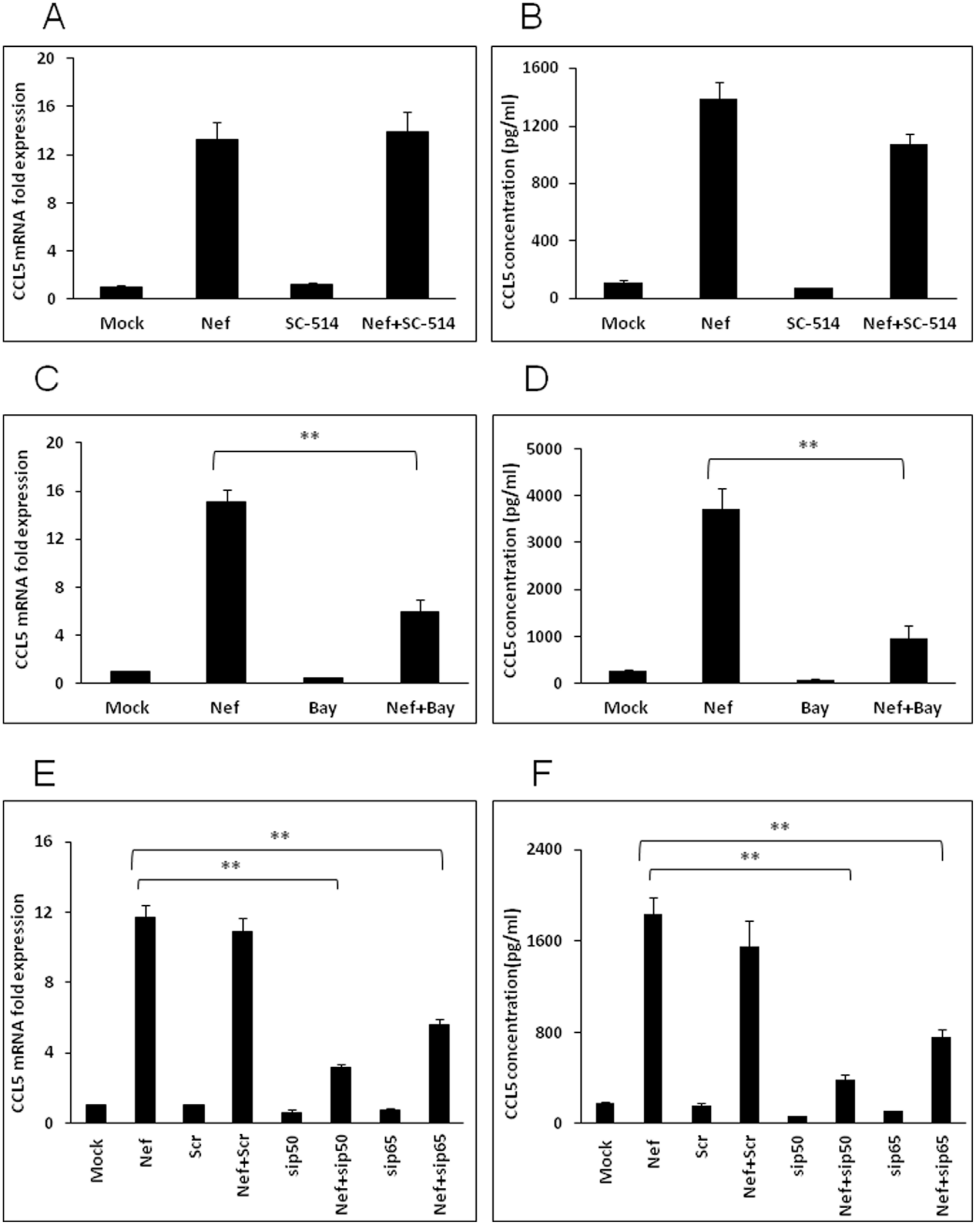
Involvement of NF-κB pathway in Nef-mediated increase of CCL5 expression. SVGA cells were treated with 20 μM SC-514 or 10 μM Bay11-7082 prior to the transfection. CCL5 mRNA (A and C) as well as protein (B and D) levels were determined at 6 and 48 h post transfection, respectively. For knockdown of p50 and p65, the cells were transfected with the siRNA followed by transfection of nef-encoding plasmid as mentioned in the materials and methods. The expression of CCL5 at the mRNA and protein levels were determined at 6 h and 48 h post-transfection by real-time RT-PCR (E) and multiplex cytokine assay (F), respectively. The values presented for mRNA are relative to the mock-transfected controls. Each of the bars represents mean ± SE of three independent experiments in triplicates. Statistical analysis was performed using one way ANOVA with the LSD post-hoc test in which * represents p-value ≤0.05 and ** represents p-value ≤0.01.

Since the NF-κB pathway is a well characterized regulator of both chemokines and cytokines, we determined the role of this transcription factor in Nef-mediated induction of CCL5. First, a specific inhibitor for IKK-2, SC-514, was added to the culture medium at a concentration of 20 μM 1 h prior to transfection. Surprisingly, it failed to abrogate the expression of CCL5 either at mRNA or at protein level (Fig. 3A–B). In separate experiments, a chemical inhibitor of both IKK1 and IKK2, Bay11-7082, was added to the culture medium at a concentration of 10 μM when the SVGA cells were seeded and 1 h prior to transfection. The expression levels of CCL5 were reduced by 60.2 ± 7.9% at the level of mRNA (6 h post-transfection) (Fig. 3C) and 74.2 ± 7.7% at the level of protein (48 h post-transfection) (Fig. 3D). To confirm these results, we used specific siRNAs to knockdown the p50 and p65 subunits of NF-κB. The siRNAs were transfected 48 h prior to transfection with the nef-expressing plasmid. The siRNAs for both p50 and p65 showed significant reduction of CCL5 by 73.1 ± 1.9% and 52.2 ± 3.1% at the mRNA level, respectively (Fig. 3E). Similarly, the CCL5 protein expression levels in the cell culture supernatant were reduced by 79.2 ± 2.9% and 58.6 ± 4.5% by p50 and p65 siRNAs, respectively (Fig. 3F).

### PI3K-Akt pathway is involved in Nef-mediated increase of CCL5 expression

**Figure 4:**
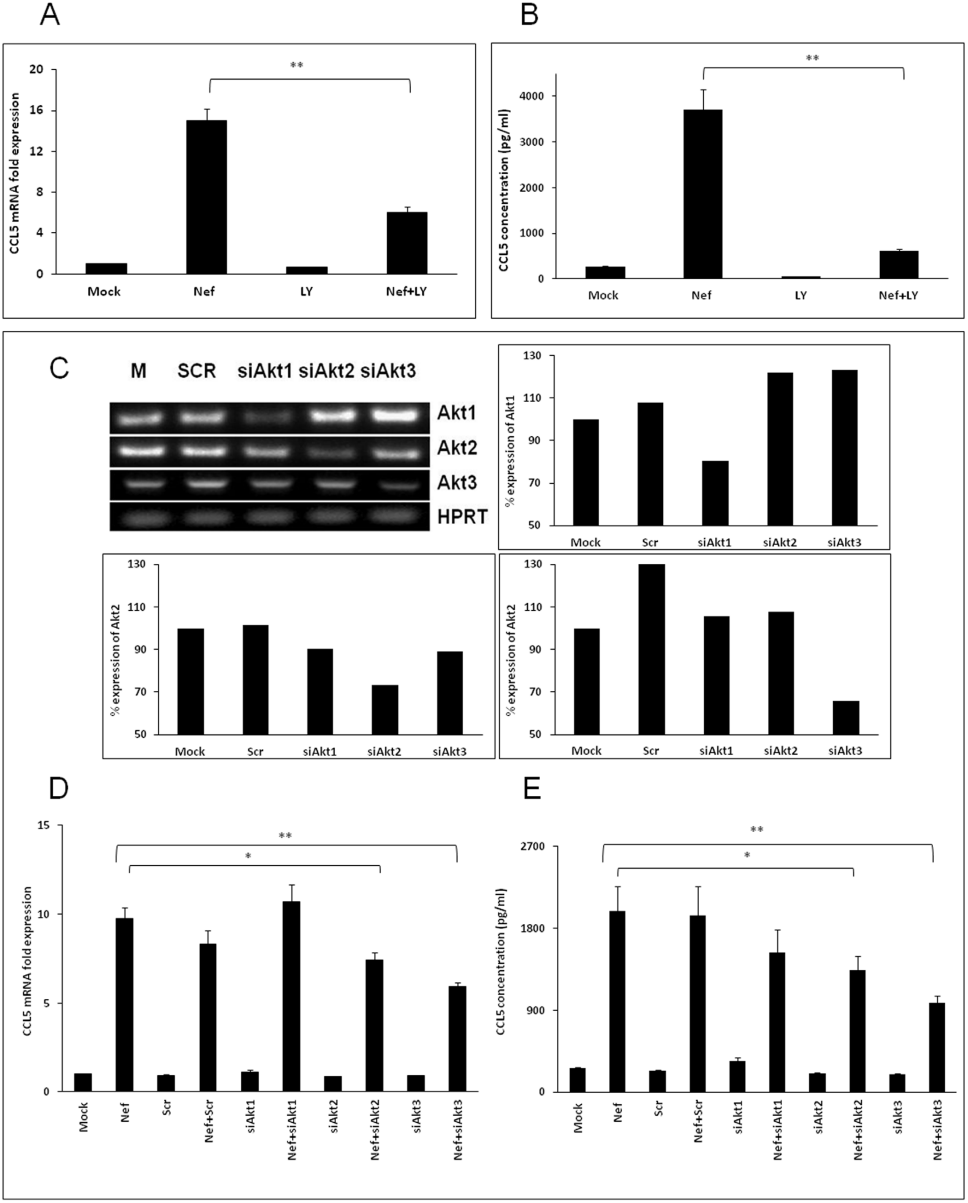
HIV-1 Nef induces CCL5 expression through the PI3K-Akt pathway. SVGA astrocytes were pretreated with 10 μM of specific PI3K inhibitor LY294002 (A–B) or transfected with siRNA against Akt1/2/3 (C–E) prior to transfection with a nef-encoding plasmid. The expression of CCL5 at the mRNA and protein levels were determined at 6 h and 48 h post-transfection by real-time RT-PCR (A and D) and multiplex cytokine assay (B and E), respectively. The values represented for mRNA are relative to the mock-transfected controls. Each of the bars represents mean ± SE of three independent experiments in triplicates. Statistical analysis was performed using a one way ANOVA with the LSD post-hoc test t in which * represents p-value ≤0.05 and ** represents p-value ≤0.01. RT-PCR for Akt1, Akt2 and Akt3 was performed to amplify the mock and scrambled controls as well as the targeted RNA. The products were then resolved by electrophoresis on a 1.5% agarose gel, stained with ethidium bromide and visualized under UV light (C).

In order to determine the upstream signaling pathways involved in NF-κB activation by Nef, we first investigated the potential role of the PI3-Akt pathway. A specific inhibitor of PI3K, LY294002, was added to culture medium at a concentration of 10 μM when SVGA cells were seeded and 1 h prior to transfection. The expression levels of CCL5 were decreased by 59.9 ± 3.7% at the level of mRNA (6 h post-transfection) (Fig. 4A) and 83.2 ± 0.6% at the level of protein at 48 h post-transfection (Fig. 4B). To further validate the possible roles of various Akt isoforms, specific siRNAs for Akt1, Akt2 and Akt3 were transfected 48 h prior to transfection with nef-expressing plasmid. The siRNAs for Akt2 and Akt3 showed significant reduction of CCL5 by 23.9 ± 5.1% and 39.2 ± 2.8% at mRNA level, respectively (Fig. 4D). The knockdown of Akt2 and Akt3 by siRNA also successfully reduced the protein level of CCL5 by 32.8 ± 8.6% and 50.6 ± 4.0%, respectively (Fig. 4E). To determine the specificity of the siRNAs for Akt isoforms, PCR amplifications of the mock-transfected and scrambled siRNA controls, as well as the targeted RNAs, were performed using isoform-specific primers. The products were then resolved by electrophoresis on a 1.5% agarose gel, stained with ethidium bromide and visualized under UV light (Fig. 4C). Amplification of HPRT (hypoxanthine-guanine phosphoribosyl transferase) was used as a loading control.

### The role of p38 MAPK in Nef-mediated increase of CCL5 expression

**Figure 5:**
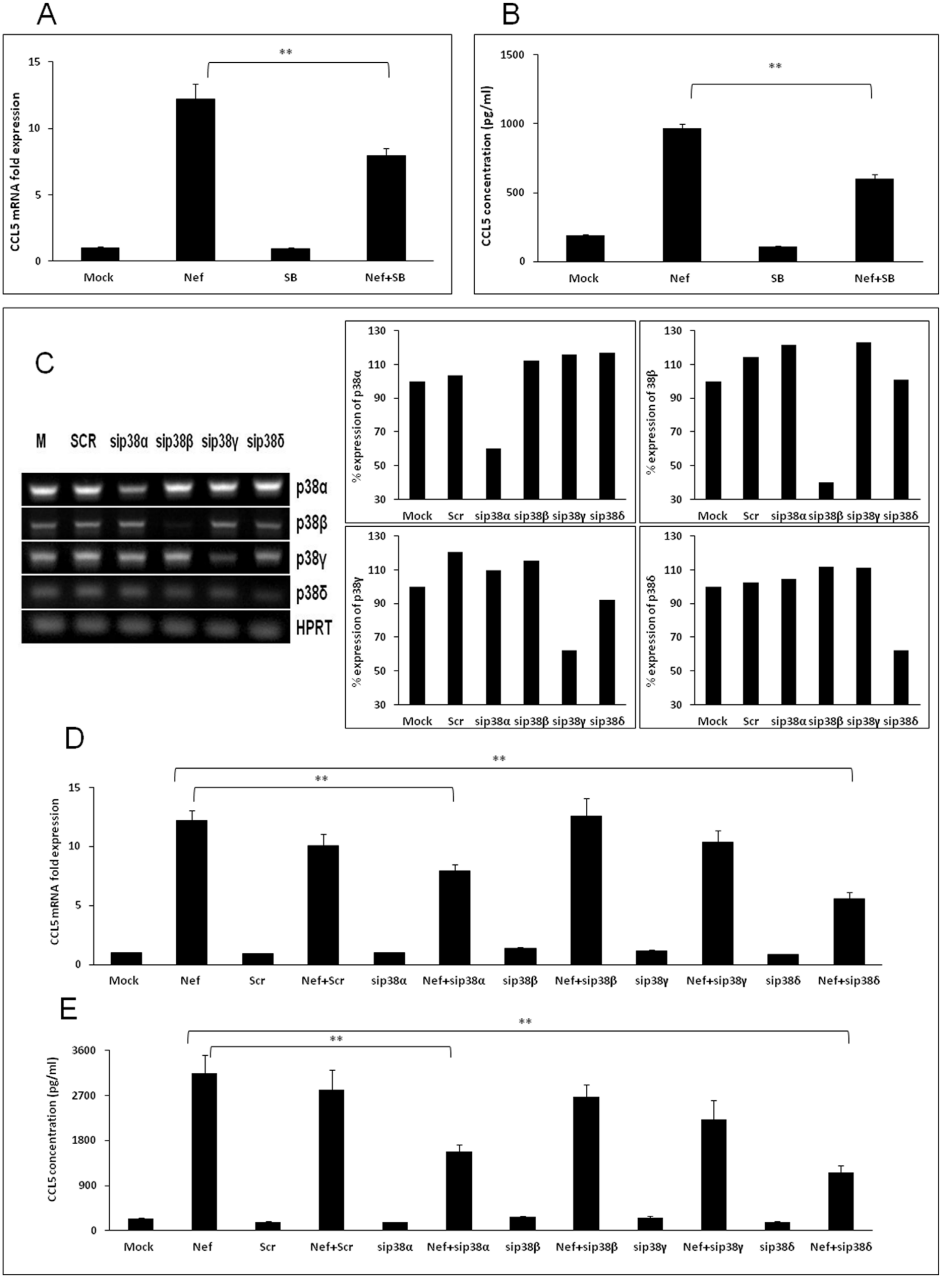
HIV-1 Nef induces CCL5 expression through the p38 MAPK pathway. SVGA astrocytes were pretreated with 10 μM of specific p38α and p38β inhibitor SB203580 (A–B) or transfected with siRNA against p38 isoforms (C–E) prior to transfection with nef-encoding plasmid. The expression of CCL5 at the mRNA and protein levels were determined at 6 h and 48 h post-transfection by real-time RT-PCR (A and D) and multiplex cytokine assay (B and E), respectively. The values represented for mRNA are relative to the mock-transfected controls. Each of the bars represents mean ± SE of three independent experiments in triplicates. Statistical analysis was performed using a one way ANOVA with LSD post-hoc test t in which * represents p-value ≤0.05 and ** represents p-value ≤0.01. RT-PCR for p38α, p38β, p38γ and p38δ was performed to amplify the mock and scrambled controls as well as silenced RNA. The products were then resolved by electrophoresis on a 1.5% agarose gel, stained with ethidium bromide and visualized under UV light (C).

In order to determine the role of the p38MAPK pathway in Nef-mediated induction of CCL5, both chemical inhibitors and siRNA approaches were utilized. First, a chemical inhibitor of p38α and p38β, SB203580, was added to culture medium at a concentration of 10 μM when SVGA cells were seeded and 1 h prior to transfection with a plasmid expressing nef. The expression levels of CCL5 were decreased by 35.3 ± 5.4% and 38.0 ± 3.4% at the level of mRNA (6 h post-transfection) and protein (48 h post-transfection), respectively (Fig. 5A–B). To further determine the potential involvement of different isoforms of p38, specific siRNAs for p38α, p38β, p38γ and p38δ were transfected 48 h prior to transfection with nef-expressing plasmid. The siRNAs for p38α and p38δ reduced the expression level of CCL5 mRNA by 29.1 ± 5.0% and 52.1 ± 4.6% (Fig. 5D), respectively. The siRNAs targeted against p38α or p38δ reduced the expression level of CCL5 protein by 49.8 ± 4.5% and 63.1 ± 5.1%, respectively (Fig. 5E). PCR was performed to amplify the mock and scrambled controls as well as the targeted RNA using p38 isoform specific primers in order to determine the specificity of the siRNAs for p38 isoforms. The products were then resolved by electrophoresis on a 1.5% agarose gel, stained with ethidium bromide and visualized under UV light (Fig. 5C). Amplification of HPRT (hypoxanthine-guanine phosphoribosyl transferase) was used as a loading control.

### The role of transcription factors C/EBP and AP-1 in CCL5 expression induced by Nef

**Figure 6:**
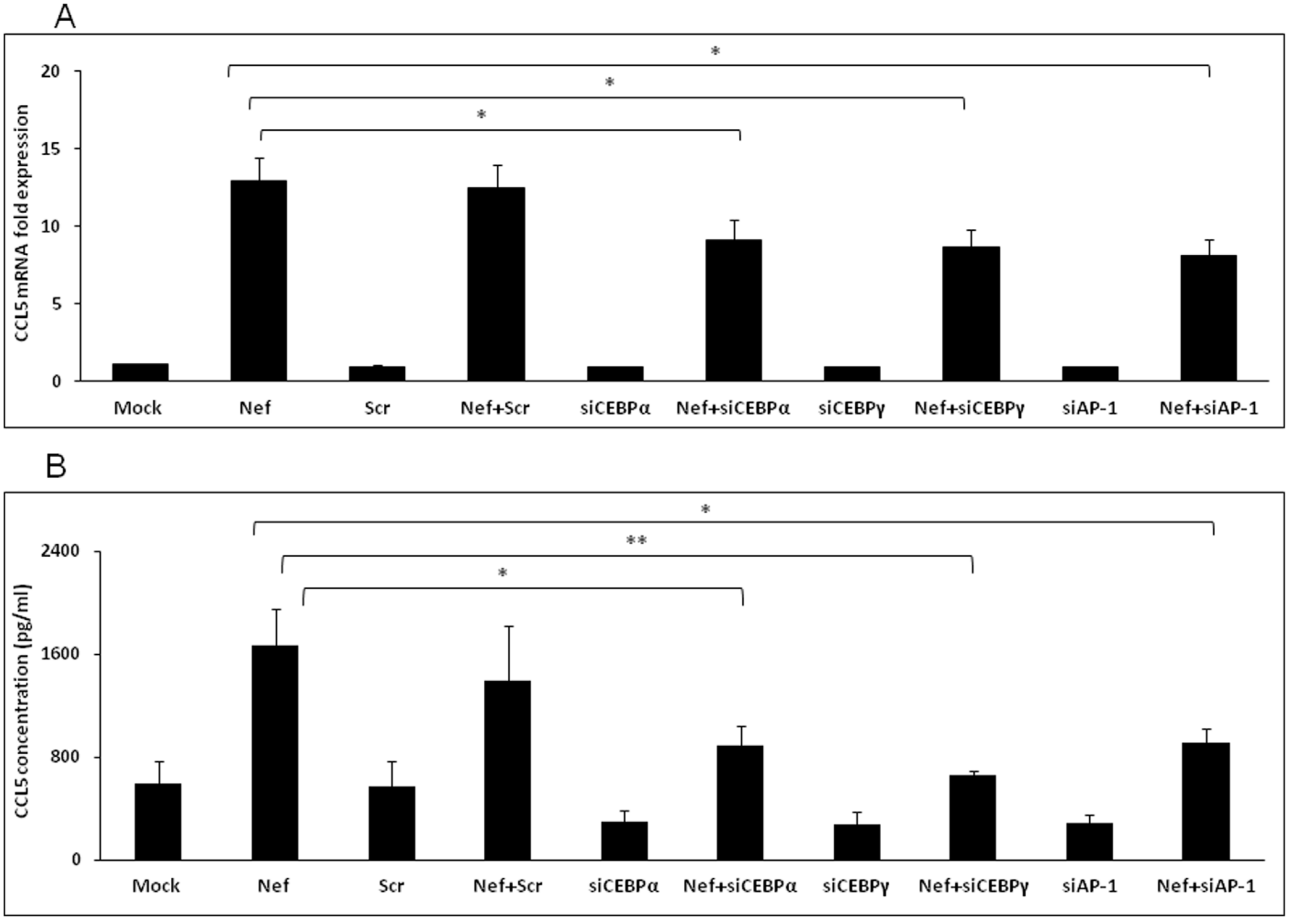
Involvement of transcriptional factors C/EBP and AP-1 in Nef-mediated increase of CCL5 expression. For knockdown of C/EBPα, C/EBPγ and AP-1, the cells were transfected with the siRNA followed by transfection of nef-encoding plasmid as mentioned in the materials and methods. The expression of CCL5 at the mRNA and protein levels were determined at 6 h and 48 h post-transfection by real-time RT-PCR (A) and multiplex cytokine assay (B), respectively. The values represented for mRNA are relative to the mock-transfected controls. Each of the bars represents mean ± SE of three independent experiments in triplicates. Statistical analysis was performed using a one way ANOVA with the LSD post-hoc test in which * represents p-value ≤0.05 and ** represents p-value ≤0.01.

Among the 6 isoforms of CCAT/enhancer binding protein (C/EBP) known in the literature^32^, C/EBPα and C/EBPγ have been shown to bind to the promoter region of CCL5 gene along with AP-1 and NF-κB^33^,^34^. Furthermore, C/EBPα, C/EBPγ and AP-1 can be activated by p38δ signaling, which was implied above to be involved in induction of CCL5 by Nef. Thus, the involvement of those transcription factors was also determined. Specific siRNAs for C/EBPα, C/EBPγ and AP-1 were transfected 48 h prior to transfection with nef-expressing plasmid. The CCL5 expression was significantly abrogated by all three siRNAs by at least 30% at both mRNA and protein levels at 6 h and 48 h post-transfection, respectively (Fig. 6A–B).

## Discussion

One of the mechanisms responsible for HIV-1-mediated neuroinflammation is the increased expression of pro-inflammatory cytokines and chemokines. One of the pro-inflammatory cytokines or chemokines that is of interest in studies focusing on neuroinflammation is CCL5. CCL5 has been shown to attract microglial cells, increase NO production and decrease IL-10 production^35^. CCL5 increases NO production^36^ and intracellular ROS^37^. Levels of CCL5 have been shown to be elevated in patients with HAD^24^,^38^ and previous work from our lab and others has demonstrated that CCL5 expression is induced in astrocytes by Tat^39^,^40^, gp120^28^ and Vpr^30^. In addition, increased CSF levels of CCL5 have been identified in HIV+ patients with some opportunistic infections of the CNS (i.e. cryptococcal meningitis) when compared with CSF levels of HIV-infected patients without the opportunistic infection^41^.

In terms of the effects of CCL5 on HIV-1, various studies had demonstrated that that CCL5 can have differential effects on viral infectivity and neurotoxicity. CCL5 concentrations of less than 500 ng/ml have been shown to be effective in blocking infectivity of macrophage-tropic strains of HIV-1^42^,^43^. However, higher concentrations of CCL5 (>0.5 ug/ml) have been shown to increase the infectivity of HIV-pseudotyped viruses regardless of the co-receptor utilized by the infecting virus^44^. In general, the higher concentrations of CCL5 have been demonstrated to provide protection from gp120-induced neurotoxicity. For example, a concentration of 20 nM (156 ng/ml) has been shown to protect primary rat neurons from gp120-induced toxicity^45^. Similar results were observed with a CCL5 concentration of 10 nM^46^. In experiments utilizing a human neuroblastoma cell line which expressed a low level of CCR5, CCL5 was neuroprotective in the range of 10–500 ng/ml^47^. Contrary to the neuroprotective effects observed, a concentration of 50 ng/ml has been demonstrated to be neurotoxic to SH-SY5Y cells transduced with CCR5^47^. However, it should also be noted that levels of CCL5 detected in CSF are in the range of pg/ml^24^,^41^.

Our results show a peak of CCL5 expression at the mRNA level 3 h after transfection of astrocytes with a plasmid encoding Nef. Although there was a decline in expression of CCL5 over time, mRNA levels remained elevated 24 h after transfection. This expression pattern was repeated when examining CCL5 protein levels in supernatants. Peak expression of CCL5 protein was observed between 24 and 48 h after transfection, when the level of CCL5 exceeded 1200 pg/ml. The increased expression of CCL5 in response to Nef transfection was also confirmed using intracellular staining. The level of expression of CCL5 we detected is below that associated with neuroprotection, but peak levels are also somewhat above the levels seen in patients with HAD and HIV+ patients with OI of the CNS^24^,^38^.

Our previous studies of the mechanisms regulating CCL5 expression by HIV-1 proteins have demonstrated a prominent role for NF-κB^28^,^30^,^39^. In addition, a binding site for NF-κB is in the CCL5 promoter and mutation of this site has been shown to reduce induction of CCL5^48^. HIV-1 Nef has also been demonstrated to activate NF-κB in various cell culture models^49^,^50^. Our studies demonstrated that Bay-11-7082, which inhibits both IKK-1 and IKK-2, inhibited induction of CCL5 by Nef. However, the IKK-2-specific inhibitor SC-514^51^ failed to inhibit CCL5 induction by Nef. This may be due to the fact that inhibition of IKK-2 by SC-514 is reversible and delays, but does abolish, degradation of IkBα Thus, while Vpr and Tat activate CCL5 through IKK-2, IKK-2 may not be required for Nef-mediated induction of CCL5. Although our results suggest that Nef may not require IKK-2 for the induction of CCL5, the results obtained with siRNAs targeted against the p50 and p65 subunits of NF-κB demonstrate that both of these subunits are required for required for induction of CCL5 by Nef.

Several lines of evidence suggested that Nef may regulate CCL5 expression through the PI3K-Akt pathway. In addition to the finding that the NF-κB transcription factor is regulated through the PI3K-Akt pathway^52^ Nef has been shown to physically interact with one of the subunits of PI3K^53^. Furthermore, some of the previous studies from our laboratory have identified the PI3K-Akt pathway as a regulator of CCL5 in astrocytes^30^,^39^. Our results showed that treatment with LY294002, an inhibitor of the PI3-Akt pathway, substantially reduced the induction of CCL5 by Nef. To further confirm the role of this pathway in the induction of CCL5, we utilized siRNAs targeted against each of the three isoforms of Akt, Akt1, Akt2 and Akt3. Whereas, siRNA targeted against Akt1 had no effect on CCL5 induction, siRNAs targeted against either Akt2 or Akt3 resulted in a substantial reduction in Nef-induction of CCL5. Interestingly, Akt3 is the most abundant Akt isoform in the cortex and hippocampus whereas in the cerebellum, Akt2 is the predominant isoform^54^. Furthermore, the Akt3 isoform has been demonstrated to play a role in various neurological processes including proper development and myelination during disease processes^54^,^55^. Finally, our previous studies regarding the effects of Tat and Vpr on astrocytes have also shown that these two isoforms are involved in the regulation of CCL5 in astrocytes^30^,^39^.

The p38MAPK pathway has been demonstrated to mediate the induction of CCL5 in brain cells in response to stimuli as rabies virus^56^, West Nile Virus^57^ and the protease-resistant domain of prion protein^58^. In addition, Nef has been demonstrated to activate p38 in as diverse macrophages^59^. In our experiments we first demonstrated that the p38MAPK inhibitor SB203580 resulted in significant inhibition of CCL5 at the level of both mRNA and protein. The chemical inhibitor SB203580 inhibits the p38α and p38β isoforms, but not the p38γ or p38δ isoforms^60^. Our experiments utilizing siRNAs targeted against all four isoforms of p38 demonstrated that p38αand p38δ were involved in Nef-mediated induction of CCL5.

In addition to the NF-κB site in the CCL5 promoter, binding sites for the AP-1 and C/EBP transcription factors have been demonstrated to bind their cognate transcription factors^33^,^34^,^48^. HIV-1 Nef has also been shown to activate the AP-1 transcription factor in the U937 monocytic cell line^49^. Using siRNAs targeted against AP-1, C/EBPα and C/EBPγ we showed that these three transcription factors are involved in mediating the induction of CCL5 by Nef.

**Figure 7:**
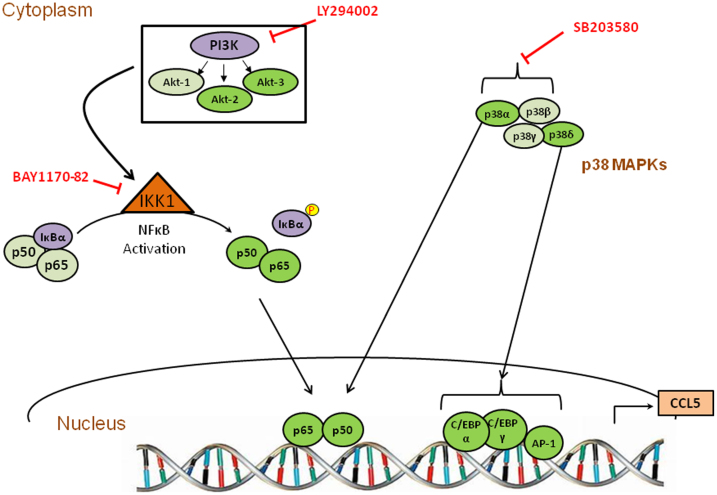
Schematic of the signaling pathways involved in CCL5 up-regulation caused by HIV-1 Nef in astrocytes. HIV-1 Nef activates PI3K-Akt and p38 MAPK pathways utilizing different subunits indicated in green (the involvement of a specific isoform is shown in brighter color and the absence is shown in pale color). These signaling molecules differentially regulate the induction of CCL5 by activating various downstream transcription factors, including NF-κB, C/EBPα, C/EBPγ and AP-1.

In summary, we characterized several pathways that are involved in Nef-mediated induction of CCL5 in astrocytes. Our results show that Nef, unlike Tat^39^, Vpr^30^ and gp120^29^, utilizes IKK1 to activate NF-κB. In terms of the PI3-Akt pathway, Nef, Tat and Vpr utilize Akt2 and Akt3. However, another difference between the induction of CCL5 by Nef as opposed to Tat or Vpr is the utilization of the p38α pathway by Nef. A depiction of the pathways involved in Nef-mediated induction of CCL5 is presented in Fig. 7. These pathways represent attractive targets for the development of pharmacotherapeutic interventions to ameliorate HIV-associated neuroinflammation.

## Methods

### Cell culture and treatment

The SVGA astrocytic cell line was a generous gift from Dr. Avi Nath^27^. The cells were maintained in Dulbecco's Modified of Eagle Medium (DMEM) (Cellgro, Manassas, VA) containing 10% FBS at 37°C in a 5% CO_2_ environment. Transfections of SVGA were performed using Lipofectamine™ 2000 as per the manufacturer's instructions (Invitrogen Inc., Carlsbad, CA) with some modifications as published in our earlier reports^28^,^29^. Briefly, 1 × 10^6^ cells were transfected with 1 μg of plasmid encoding Nef (p96ZM651 nef-opt) (AIDS Research and Reference Reagent Program) using 4 ul Lipofectamine in 1 ml serum-free media. After 5 h, transfection medium was replaced by fresh DMEM containing 10% FBS. Cells were then harvested at the indicated time for RNA or protein isolation. SC-514 (IKK 2 inhibitor), Bay11-7082 (IKK inhibitor), LY294002 (PI3K inhibitor), and SB203580 (p38 MAPK inhibitor) were added 1 h prior to transfection of HIV-1 Nef. All inhibitors were obtained from Cayman Chemicals (Ann Arbor, MI).

### Real time RT-PCR and multiplex cytokine assay

Total RNA was extracted using RNeasy mini kits (Qiagen, Valencia, CA) as described in the manufacturer's protocol. Real time reverse transcriptase polymerase chain reactions (RT-PCR) using Bio-Rad iCycler was performed to determine the mRNA expression levels. The reaction conditions included reverse transcription at 50°C for 30 min, denaturation at 95°C for 15 min, followed by amplification for 45 cycles (95°C for 15 sec, 54°C for 30 sec, and 76°C for 15 sec). The CCL5 primers are 5′ ACC AGT GGC AAG TGC TCC A-3′ for forward and 5′ ACC CAT TTC TTC TCT GGG TTG GCA-3′ for reverse (Integrated DNA Technology, Coralville, IA). Separate hypoxanthine-guanine phosphoribosyl-transferase (HPRT - house-keeping gene) amplification was used to normalize gene expression. The data was analyzed using the equation 2^−ΔΔCT^ method. The primer sequences and PCR conditions for p38 and Akt isoforms used in this study and the annealing temperatures were based on our previous study^30^.

Cell culture supernatants were collected and the cytokine protein concentrations were analyzed by a multi-cytokine bead assay system (Bio-Rad, Hercules, CA) according to the manufacturer's instructions. The protein expression was measured using Bio-Plex Manager 5.0 software and 5PL standard curve.

### Transfection with siRNAs

NF-κB, and p38 α/β/γ/δ siRNAs (Ambion, Austin, TX), as well as siRNAs against C/EBPα, C/EBPγ, AP-1, and Akt (1/2/3) (Thermo Fisher Scientific, Pittsburgh, PA) were transfected 48 h prior to nef transfection. 2 × 10^5^ cells in each well were transfected with 50 nM siRNA in serum-free media. After 24 h, the transfection medium was replaced with fresh DMEM supplemented with 10% FBS. Cells were then trypsinised 8 h later and re-seeded at a density of 2.5 × 10^5^ per well in a 12-well plate. Nef transfection was then performed the next day as described above and cytokine levels were determined at different time points. Transfection with 50 nM scrambled siRNA was used as the negative control.

### Agarose gel electrophoresis

The amplified products for Akt and p38 isoforms from PCR were electrophoresed on 1.5% agarose gel for 1.5 h at 100 V. The gel was stained with 0.005% Ethidium Bromide, and the results were obtained using the Alpha Innotech FluorChem® for 8 ms under UV light.

### Immunocytochemistry

SVGA cells were seeded on 1.5 mm cover slips in a 6-well plate followed by either mock transfection, or transfection with a plasmid encoding HIV-1 Nef. After 5 h, transfection medium was replaced by fresh DMEM containing 10% FBS with addition of 3 μM GolgiStop™ (BD Biosciences, San Jose, CA) in order to prevent the release of CCL5. Twelve hours later, the cells were fixed with 1:1 ice-cold methanol:acetone for 20 min at 20°C. Then the cells were washed 3 times with PBS followed by blocking with 1% BSA in PBS with 0.1% Triton for 30 min at room temperature. All the antibodies were diluted in 1% BSA in 0.1% PBS. After blocking, the cells were incubated with a cocktail of rabbit anti-RANTES antibody (1:200) (Thermo Fisher Scientific, Rockford, IL) and mouse anti-GFAP antibody (1:1500) (Abcam, Cambridge, MA) overnight in a humidified chamber. After washing three 5 min with PBS with 0.1% Triton, the cells were incubated in the dark for 1 h at room temperature with an anti-mouse antibody conjugated with Alexa Fluor 555 (1:1500) and an anti-rabbit antibody conjugated with Alexa Fluor 488 (1:1500) (Cell Signaling, Danvers, MA). Finally, after another three washes, the coverslip was transferred onto a slide with 10 μl of Vectashield mounting medium with DAPI (Vector Laboratories, Burlingame, CA). The fluorescence microscopy was performed using a Leica TCS SP5 II Laser Scanning Confocal microscope. The images were captured using a 40× zoom lens.

### Statistical analysis

A one way ANOVA with a least significant difference ((LSD) post-hoc test was used to calculate the statistical significance in all the experiments. The results showed mean ± standard error (SE) of at least three individual experiments. P-value of ≤0.05 was considered to be statistically significant.

## Author Contributions

X.L., A.S. and M.G. performed all the experiments presented, performed the data analysis. X.L. also contributed to the writing of the manuscript. P.S.S. edited various parts of the manuscript. M.F. helped with experimental design. A.K. designed the overall project, supervised the data analysis and contributed to the editing of the manuscript. All authors have reviewed the manuscript.
